# Vision System for a Forestry Navigation Machine

**DOI:** 10.3390/s24051475

**Published:** 2024-02-24

**Authors:** Tiago Pereira, Tiago Gameiro, José Pedro, Carlos Viegas, N. M. Fonseca Ferreira

**Affiliations:** 1 Polytechnic Institute of Coimbra, Coimbra Institute of Engineering, Rua Pedro Nunes—Quinta da Nora, 3030-199 Coimbra, Portugal; tmsp1998pereira@gmail.com (T.P.); tiagocostagameiro@gmail.com (T.G.); 2 ADAI (Associação para o Desenvolvimento da Aerodinâmica Industrial), Department of Mechanical Engineering, University of Coimbra, Rua Luís Reis Santos, Pólo II, 3030-788 Coimbra, Portugal; josepedropm@hotmail.com (J.P.); carlos.viegas@uc.pt (C.V.); 3 GECAD—Knowledge Research Group on Intelligent Engineering and Computing for Advanced Innovation and Development, Engineering Institute of Porto (ISEP), Polytechnic Institute of Porto (IPP), 4200-465 Porto, Portugal

**Keywords:** vision system, image processing algorithms, autonomous mobile robot, YOLOv5, object detection

## Abstract

This article presents the development of a vision system designed to enhance the autonomous navigation capabilities of robots in complex forest environments. Leveraging RGBD and thermic cameras, specifically the Intel RealSense 435i and FLIR ADK, the system integrates diverse visual sensors with advanced image processing algorithms. This integration enables robots to make real-time decisions, recognize obstacles, and dynamically adjust their trajectories during operation. The article focuses on the architectural aspects of the system, emphasizing the role of sensors and the formulation of algorithms crucial for ensuring safety during robot navigation in challenging forest terrains. Additionally, the article discusses the training of two datasets specifically tailored to forest environments, aiming to evaluate their impact on autonomous navigation. Tests conducted in real forest conditions affirm the effectiveness of the developed vision system. The results underscore the system’s pivotal contribution to the autonomous navigation of robots in forest environments.

## 1. Introduction

The quest for technological solutions capable of handling challenging environments has driven significant advancements in the fields of mobile robotics and computer vision. Efficient employment of computer vision presupposes meeting certain requirements such as good lighting and appropriate brightness in the environment being worked on. Thus, applying cameras with good definition allows for capturing images to proceed with their processing. It is common to preprocess images, such as their segmentation, which will provide additional information, allowing for the extraction of valuable and critical information for their classification, fulfilling the objective of obtaining the desired output from a system [1].

Using different types of cameras, it is possible to implement detailed visual perception of a particular environment, thereby acquiring capabilities to be applied in the domain of autonomous driving [2]. For perceiving a specific environment, various functions are involved, such as recognition, object extraction, and segmentation, with cameras. This type of method involves the use of deep learning algorithms such as single shot detector (SSD) [3], region-based convolutional neural networks (R-CNN) [4], and you only look once (YOLO) [5].

Specifically, the ability of robots to navigate autonomously in forest environments requires systems and algorithms that provide safety during their autonomous navigation, making computer vision a fundamental topic for decision making. This type of decision making can be scaled in different aspects in a forest environment, depending on the different types of objects that may be present. For example, detecting static objects in a forest environment can be considered an object by which a certain robot can steer clear; however, dynamic objects such as people or animals must consider the unpredictability and thus immobilize the operation of a forest robot.

This article proposes an auxiliary technological system for the autonomous navigation of a robot in a forest environment, grounded in the integration of computer vision and an image processing algorithm (in this case, the YOLOv5 algorithm), to enable a robot to move safely in forest environments. The goal is to endow the robot with the ability to make real-time decisions based on accurate interpretation of visual information, allowing for the detection and identification of obstacles, and, based on these procedures, change its operational state. We intend to present in detail the architecture of the vision system, emphasizing the purpose of each visual sensor and the strategies used, which will be experimentally validated under real field conditions, thus demonstrating the impact of this vision system during the autonomous navigation of a forest robot. Additionally, parallel to this, we aim to train two datasets for detecting tree trunks and vegetation in order to analyze the impact, they may have on the navigation of the forest robot in question. Therefore, the main contributions of this work include:

- Development of an architecture for a vision system that adapts to forest environments, emphasizing the importance of applying the proposed sensors.
- Development of an algorithm that ensures safety during the autonomous navigation of the robot by detecting people and measuring distances to them.
- Training of two datasets for object detection in forest environments and analysis of the impact these datasets can have during robot navigation.

## 2. Related Work

Computer vision has played a crucial role in image processing and the integration of systems involving the acquisition, transmission, processing, and understanding of visual information from images. This one has a wide range of applications in robotics, such as planetary exploration. For instance, the Institute of Robotics and Mechatronics of the German Aerospace Center (DLR) developed a prototype called the Lightweight Rover Unit (LRU) dedicated to planetary exploration. The LRU uses a stereo camera to identify unknown objects [6]. Another application is in search and rescue, where the Centre for Automation and Robotics (CAR) at the Polytechnical University of Madrid implemented a method based on thermal image processing and the application of convolutional neural networks (CNN) to identify victims in post-disaster environments (PDE). This is achieved with the assistance of a robotic system that moves through a legged system [7]. Visual Simultaneous Localization and Mapping (Visual SLAM) is another use of computer vision in robotics. Using images of an environment allows a robot to self-localize on a given map and participate in its construction [8].

In agriculture, computer vision on robotic platforms has been gaining increasing relevance and experiencing significant development. Within agriculture, vision has been applied to various operations such as weed detection, crop inspection, harvesting, spraying, and more [9]. The integration of machine vision technologies in agricultural robots is pivotal for addressing navigation challenges, enhancing adaptability, and ensuring the efficiency of smart agriculture in meeting the escalating demands of a growing global population [10]. Finally, in forestry, computer vision has made significant strides, as seen in projects such as SEMFIRE. This project aims to develop a robotic platform for reducing forest fuel. By employing computer vision and applying CNN, it becomes possible to identify and characterize the environment (trees, animals, and people) as well as the vegetation to be removed [11]. Reference [12] underscores experiments conducted in the UK aimed at monitoring forests for economic growth and climate regulation. The experiments utilized a drone equipped with camera sensors, including thermal and RGB cameras, and employed the YOLOv5 algorithm for recognition.

When it comes to object detection algorithms, YOLOv5 has proven to be one of the most widely used applications in forest environments as well as in agriculture. In the work conducted by Wang H. et al., the YOLOv5 algorithm is utilized to detect lychees along with the Intel RealSense D435i camera to automate and optimize lychee harvesting [13]. Similarly, Hofinger P. et al. used the YOLOv5 algorithm to monitor tree diseases where images were captured by a UAV. Results obtained from this study revealed that the architecture imposed by the YOLOv5 algorithm is suitable for the proposed task, thus enabling the detection of damaged black pines with a 95% confidence interval [14]. Lastly, the work carried out by Niu K. et al. demonstrated that the use of YOLOv5 proves advantageous for fire detection. Thermal images captured from a UAV are utilized for this purpose [15].

Compared to other object detection algorithms, YOLOv5 has proven to be one with higher performance. For instance, in the work conducted by Naftali M. et al., a comparison of five object detection algorithms in outdoor environments, such as SSD MobileNetv2 FPN-lite 320 × 320, YOLOv3, YOLOv4, YOLOv5l, and YOLOv5s, was performed. It was found that the algorithm that showed the most efficacy was YOLOv5s, thus revealing an advantage for this type of application [16]. The authors of [17] conducted the detection of different types of objects in forests using the algorithms Fast R-CNN, YOLOv3 SSD, and YOLOv5, where it was found that YOLOv5 proved to be lighter and faster in object detection, achieving better accuracy compared to the other algorithms.

## 3. Methodology and Algorithms

### 3.1. System Description

**Figure 1:**
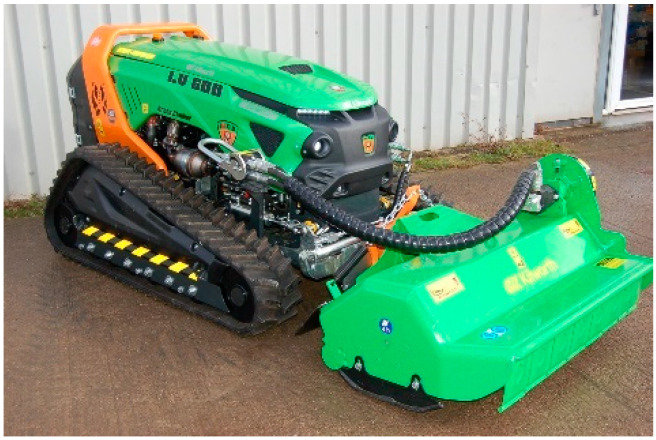
Forestry Machine MDB LVB600 PRO.

The robotic platform used for this study is a Green Climber LV 600 PRO forest machine by MDB (manufactured by MDB, Fossacesia, Italy), as depicted in Figure 1. It is a remotely controlled tractor designed for vegetation cutting in dense forest environments.

For its control and sensory perception, an architecture named Sentry was defined, consisting of a “plug and play” sensor box adapted to the specifications of this and other forest machines. This system incorporates a set of sensors that enable different levels of autonomy, defining various subsystems, each with its own architecture, to provide autonomous navigation for the robot. The Sentry system is mounted on the rear of the tractor, connected to the forest machine, making it possible to control the vegetation-cutting equipment.

**Figure 2:**
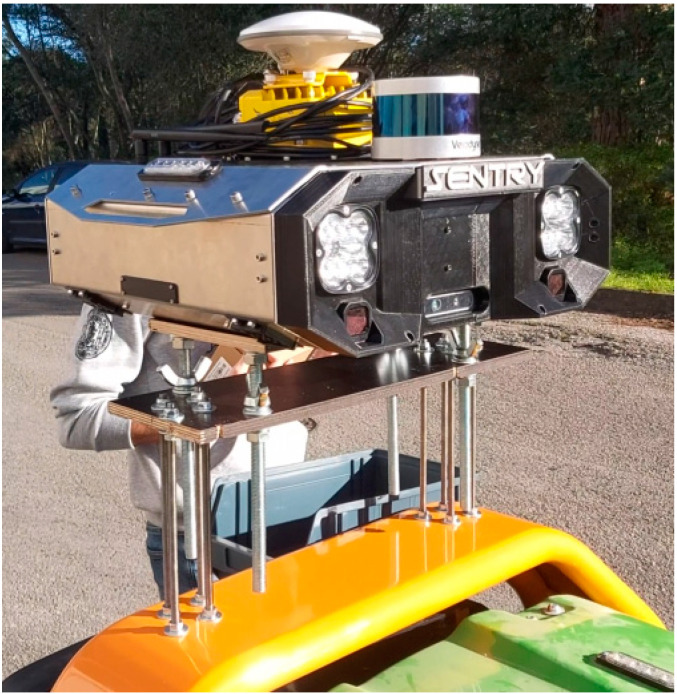
Sensory system (SENTRY).

The sensory system has a sensor architecture. Equipped with various sensors, including high-resolution cameras, LIDAR, a Global Navigation Satellite System (GNSS) unit, IMU, and magnetometer, the Sentry can capture real-time information about the surrounding forest environment. The system is shown in Figure 2.

This diverse data combination provides a comprehensive and accurate view of the terrain, allowing for obstacle detection [18]. The processing unit consists of a minicomputer called NVidia Jetson Xavier Nx, which has notable features enabling efficient execution of algorithms, including neural networks, while simultaneously processing data from high-resolution sensors.

### 3.2. Vision System Architecture

**Figure 3:**
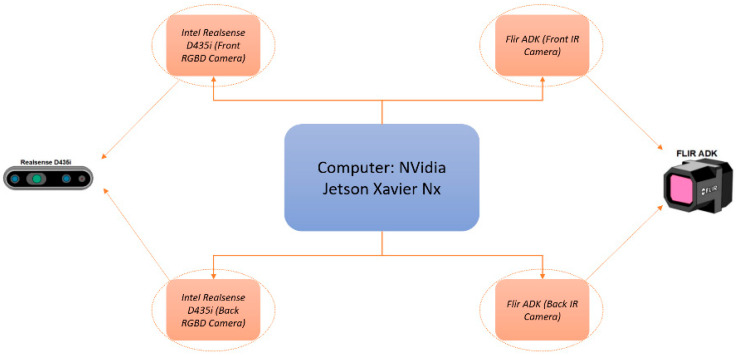
Composition of the sensory elements of the vision system.

The autonomous navigation of a forestry machine such as the MDB LV600 PRO must consider all the safety issues, such as the detection of people and other obstacles near the machine. It is necessary to equip the Sentry with a vision system that allows the detection of these types of obstacles. This architecture consists of four cameras, where two are RGBD and the other two are thermal, as shown in Figure 3.

The choice of RGBD cameras went through a rigorous and comparative process, considering potential cameras such as the Intel RealSense D435I (manufactured by Intel Corporation, Aloha, OR, USA; Hillsboro, OR, USA) and the ZED 2i (manufactured by Stereolabs, San Francisco, CA, USA; New York, NY, USA and Paris, France). This selection was based on the work carried out by Vladimir Tadic et al. in [19], where Table 1 presents a comparative study between these cameras.

The ZED 2i camera demonstrates superior performance in terms of features and functionalities for robotics applications, as indicated in Table 1. However, due to the development stage of the Sentry system, the more cost-effective option, Intel RealSense D435i, was chosen. This camera is designed for depth sensing in various applications, including computer vision. It has a wide field of view (H × V × D) of 91° × 65° × 100° for the RGB camera, which reduces blind spots in the depth map. The corresponding depth sensor has an FOV of 85° × 58° × 90°. The impressive depth-sensing capability of the D435i makes it suitable for a variety of uses, such as robotics, virtual reality, and 3D scanning, among others. It stands out for its accuracy in in-depth measurement, resulting in more precise mappings and enhanced object localization. With a depth range of up to 10 m, this camera offers a wide range of applications [19].

The FLIR ADK thermal cameras, developed by the FLIR System, use infrared sensors to capture thermal images of the surrounding environment. Specifically designed for object detection and temperature information, the FLIR ADK excels in providing data in conditions of low visibility, such as darkness, fog, smoke, or other common adverse scenarios [20]. In addition to the mentioned capabilities, the FLIR ADK can be combined with other sensors, such as RGB cameras and LIDAR sensors, to create a comprehensive vision system of the environment around the machine. This sensor fusion provides a more accurate perception of the scenario, enabling object identification and enhancing safety during vehicle navigation [20]. The need to integrate FLIR ADK cameras arises in adverse situations, such as the presence of dust, common in forestry operations. In such environments, the introduction of dust can impair the performance of certain sensors, such as RGBD cameras, making infrared cameras a valuable resource for these situations [21]. In the Sentry, the RGBD cameras will be located at the front and back of the box, while the thermal cameras will be located at the front of the box. The primary goal of this vision system is to provide increased safety throughout the operation of the LV600 Pro; thus, sensor redundancy is desired, to cover all possible scenarios (dust, debris, smoke, multiple lighting conditions, etc.).

### 3.3. Interaction between Subsystems

To provide a robust and modular framework for control and coordination among all subsystems, the ROS (robot operating system) platform was employed. ROS enables and facilitates communication and synchronization among various robotic components through its distributed architecture, allowing precise and efficient execution of all components. The integration of ROS into the Sentry system allows for data acquisition from the environment, enabling the robot to adapt its capabilities to face a variety of environmental challenges in forestry settings.

The interaction between ROS and vision systems goes beyond data transfer; it extends to dynamic decision making as the robot navigates a specific trajectory. Using an image processing algorithm, this vision system can continuously analyze the environment and provide critical information to ROS. This capability enables the robot to maintain a safe trajectory, even in complex and unpredictable situations.

### 3.4. YOLOv5—The Object Detection Algorithm

The precise and efficient detection of objects in images within a given scenario is crucial, spanning from autonomous vehicles to security systems and robotics. The work conducted placed more emphasis on YOLOv5, the fifth version of the renowned you only look once (YOLO) architecture. This algorithm belongs to the YOLO family due to its efficiency and real-time object detection speed. YOLOv5 represents the fifth generation of this series, offering significant improvements over its predecessors [22]. Its preference in the scientific community has stood out due to its excellent performance in complex and noisy data contexts and its simplicity of use with popular programming languages such as Python.

**Figure 4:**
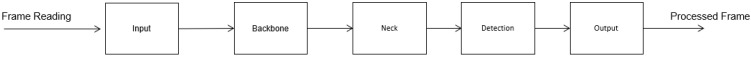
Structure of the YOLOv5 algorithm (Based on [23]).

The architecture of YOLOv5 comprises five sections, as depicted in Figure 4. The input block divides the image into sections, with each section responsible for detecting a specific region of the image. The backbone extracts image features, while the neck, an intermediate part of this architecture, performs fusion to enhance object detection. The detection section assigns a specific class to the detected objects, and finally, the output generates detection results such as class, confidence, location, and size [23].

This system uses a convolutional neural network to extract and organize image features at various levels and then combines these features, preparing them for the prediction step [23]. YOLOv5 operates in two distinct phases: training and inference. During training, the model is exposed to an extensive set of images containing the objects to be detected. In the inference phase, the previously trained model is fed with new images to perform the detection of the previously taught objects. YOLOv5 processes the image in a single pass through various convolutional layers, resulting in quick and highly reliable detection [22].

Compared to its predecessor, YOLOv4, the YOLOv5 algorithm shows significant improvement. Its accuracy has been enhanced, especially for smaller-sized objects, while maintaining computational efficiency. The fifth version of YOLO (YOLOv5) introduces improvements in accelerating the inference process, making it more suitable for real-time applications [24]. When compared to the Faster R-CNN algorithm, which exhibits excellent accuracy but operates in multiple successive steps [22], YOLOv5 stands out for its computational efficiency. While Faster R-CNN functions in several successive steps, YOLOv5 uses a single-pass approach, analyzing the image only once [22]. This provides faster inference, making YOLOv5 more suitable for applications requiring low latency.

In comparison to the SSD algorithm, YOLOv5 surpasses it in terms of accuracy while maintaining similar efficiency. YOLOv5’s ability to analyze an image at once, instead of dividing it into multiple regions [25], contributes to more precise and efficient detection. This is especially crucial when rapid and accurate detection is essential. Thus, YOLOv5 emerges as an ideal choice for object detection, offering a combination of accuracy and computational efficiency.

### 3.5. Vision System Tasks

The Sentry vision system, as indicated, comprises two RGBD cameras and two thermal cameras. The work carried out on the Sentry vision system was divided into three tasks, namely:

- Verification of the operation of YOLOv5 with NVidia Jetson Xavier Nx.
- Development of an algorithm that integrates object detection with their measurement.
- Training of a dataset for object detection in a forest environment.

#### 3.5.1. Object Detection and Distance Measurement

**Figure 5:**
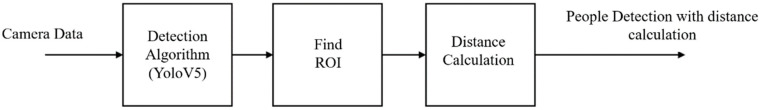
Vision system model.

The work carried out on this topic focused on the analysis of data from RGBD cameras. An algorithm was implemented for object identification and distance measurement. Emphasis was given to the detection of people, and the algorithm was used to measure distances to obstacles in the environment to change the robot’s state. For this specific case, it was decided that when the robot is executing a particular trajectory and detects a person within a certain distance, the robot should immobilize itself. This method was proposed because we are dealing with a forest grinding machine, and when it performs the vegetation grinding function, it sometimes projects forest residues. Therefore, immobilizing the machine along with all its functions becomes an important aspect for the safety of both the machine and the surrounding environment during its navigation. The block diagram presented in Figure 5 illustrates the functioning of the vision system incorporated into the Sentry system.

The distance measurement is carried out through RGBD cameras (Intel RealSense). The RGB cameras capture images of a given environment, while the depth camera measures the distance of objects using a time-of-flight (ToF) method. The Intel RealSense depth sensors are equipped with three camera lenses, including an infrared (IR) camera, an RGB camera, and an infrared laser projector. Therefore, these depth sensors are referred to as active devices because they incorporate an IR laser projector to enhance depth measurement. By combining the three lenses, the perception of infrared light reflected by the object in front of the lenses is achieved. To calculate depth, Intel RealSense sensors use stereo vision by combining data from the sensor on the right side, one on the left side, and the infrared laser projector. This projector emits invisible rays that improve the accuracy of depth data in environments with limited textures. The sensors capture the scene and send information about the real image to the microprocessor. Based on this data, the processor determines depth values for each pixel in the recorded image. This involves correlating the values obtained with the right camera with the image derived from the left camera. The resulting depth data from each pixel processed in this way forms the depth image [19].

**Figure 6:**
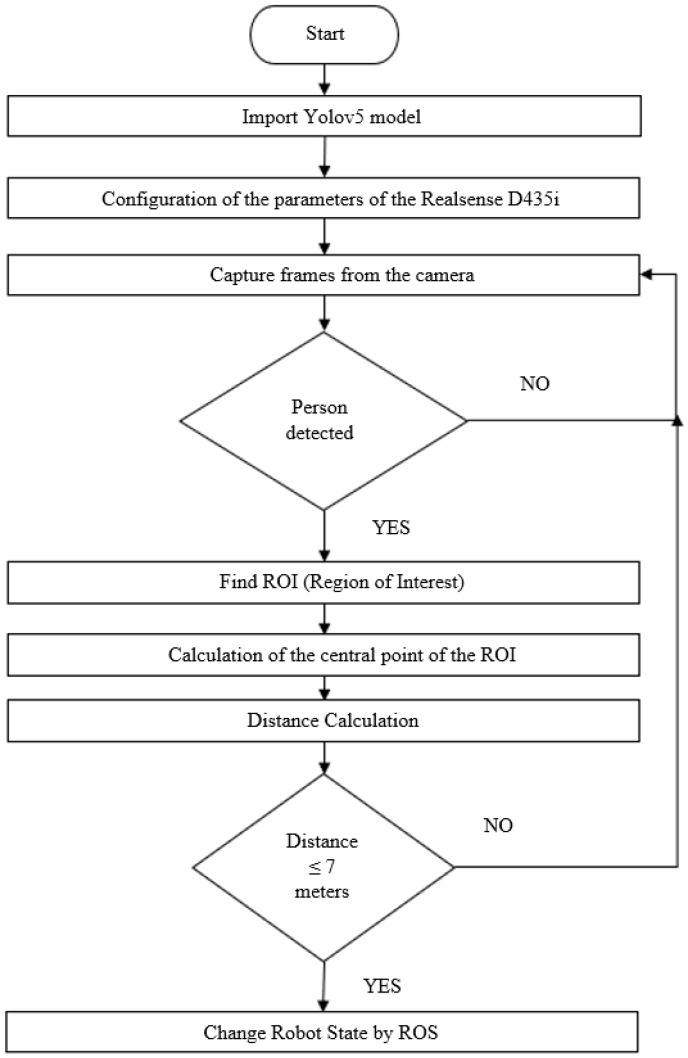
Process for detecting people with distance.

To perform distance measurements, the pyrealsense2 library associated with Intel’s RGBD cameras was utilized, and its source code is available at [25]. The pyrealsense2 library facilitates data processing to improve the quality of captured information using filters and preprocessing methods. Thus, it is necessary to develop an algorithm that allows connecting object detection by YOLOv5 with distance measurement using pyrealsense2. This algorithm can be observed in the flowchart in Figure 6.

The process begins with importing the model trained by YOLOv5, followed by configuring the parameters of the Intel Realsense camera. Once this step is completed, the camera acquires images. If people are detected with a confidence rate of 70% (provided by YOLOv5 each time it detects an object), a location identification process is initiated using the region of interest. This involves finding the center of this specific area. Based on this central point, the distance measurement is performed. For this work, it was established that if the distance is less than or equal to 7 m, the system triggers an emergency stop. This means that a Boolean signal is sent to the Sentry system, which changes the state of the machine and puts it in a safety stop state. This Boolean signal needs to be a topic in ROS so that when the machine is in autonomous navigation in the future, it immediately changes its state.

#### 3.5.2. Training Datasets for Forest Environments

To perform object detection in forest environments, a dataset was trained, considering categories such as tree trunks and forest fuel material. At this stage, the goal is to identify these classes using a convolutional neural network (CNN) model. Existing and suitable datasets for the training process were selected. Table 2 provides a summary of the number of images and their distribution in each of the chosen datasets.

For the detection of tree trunks, the Trees_dataset, created by Silva et al. [26], where the source code is available at [27], was utilized. This dataset consists of 2895 images, including 2029 RGB images and 866 thermal images. In the detection of forest fuel, the Vegetation_detection dataset, created by Mendes et al. [28], where the source code is available at [29], was employed, comprising 199 RGB images. To assess the performance of the training of each dataset, the mAP50, mAP50:95, precision, and recall metrics were utilized. The mAP50 metric evaluates how accurate the model is in detecting objects when there is an overlap of at least 50% with the true bounding box. The mAP50:95 metric provides a more comprehensive perspective on how well the model performs over a wide range of overlap levels. The precision metric is a measure that indicates how many objects were correctly detected concerning the total detections made by the model, thus assessing the model’s ability to avoid misidentifications. Finally, the recall metric indicates how many true objects were correctly identified concerning the total number of true objects present in the image, thus evaluating the model’s competence in identifying most of the objects present in the image.

## 4. Experimental Tests

To assess the performance of the vision system algorithm to be integrated into the robot, three distinct tests were conducted:

- The first test, conducted in the laboratory, aimed to evaluate the capability of the YOLOv5 algorithm to function on the Jetson Xavier Nx, using two thermal cameras and the RGB camera from Intel, along with the depth camera.
- The second test was performed in a forested area, with the forestry machine operational, to evaluate the performance of the developed algorithm, as shown in Figure 6.
- The third test aimed to demonstrate the training of a dataset for the identification of tree trunks and vegetation using YOLOv5.

### 4.1. YOLOv5 Performance with NVidia Jetson Xavier Nx

**Figure 7:**
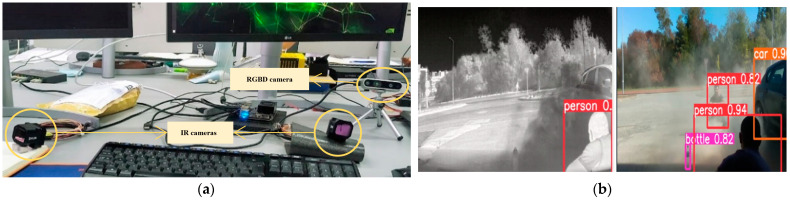
Test to verify the operation of YOLOv5. (**a**) Setup with two thermal cameras, one RGB camera, and one depth camera; (**b**) YOLOv5 response.

Regarding the first test, as shown in Figure 7, the setup with the cameras and the operation of YOLOv5 in each of them is presented. For this test, the official YOLOv5 model was used, specifically the yolov5s version. The main objective of this test was to verify the performance of the YOLOv5 algorithm with the Nvidia Jetson Xavier Nx in order to evaluate the Jetson’s performance in object detection.

It was observed that object detection at a rate of 100 ms allows for effective obstacle detection when the machine is in autonomous mode. This validates the operation of YOLOv5, where relevant information can be extracted for use when the machine is autonomously navigating a trajectory.

### 4.2. Validation of the Proposed Algorithm for Object Detection and Distance Measurement Fusion

**Figure 8:**
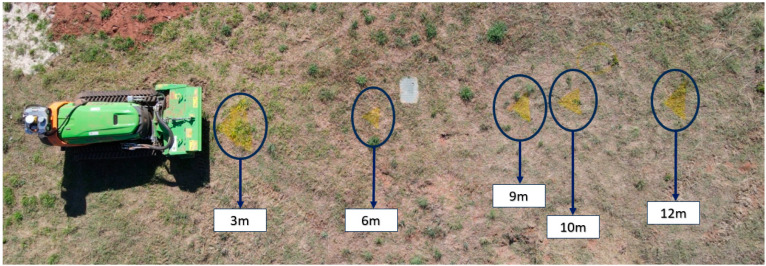
Distances defined for comparing real data with camera data.

In order to verify the performance of the proposed algorithm in Figure 6, an experimental test was conducted, specifically, the verification of distance measurement when a person is detected (this information comes from YOLOv5, as demonstrated in the test conducted in Section 4.1). To conduct this test, markers were placed on the ground at strategic distances, as illustrated in Figure 8. A person occupied each of these positions at the markers, providing real distance data, which were then compared with the camera readings. The markers were placed at intervals of 3, 6, 9, 10, and 12 m. The variance between the camera data and the actual values obtained from these markings was calculated.

**Figure 9:**
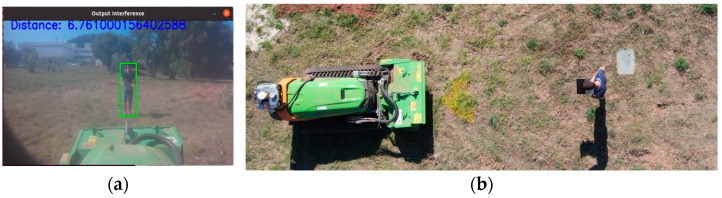
Test to verify the measurement from the camera. (**a**) Procedure for one of the stages of testing the vision system algorithm in the field. (**b**) The person located 6 m from the front camera of the Sentry.

To validate the setup in the field, a person moved to each of the strategic marks, staying at each one for approximately 3 s, and thus, approximately 20 points were collected for each position. Figure 9 shows a person’s position at one of the marks, and the vision system performed detection with distance measurement.

Table 3 presents a comparison between real measurements and those provided by the camera. There is a discrepancy between real measurements and the measurements taken by the vision system. These results may be influenced by vibrations transmitted by the forestry machine, generated by the combustion engine, which can in some way affect the performance of the cameras. However, it is observable that as the distance increases, the variance also increases. Despite this, the obtained values seem promising.

### 4.3. Benefits of the Proposed Dataset for a Forest Environment

This test aims to demonstrate the importance of the Trees_dataset and Vegetation_detection datasets for detecting trunks and vegetation in a forest environment. The validation of these two datasets involved two stages:

- Data collection.
- Results obtained from training.

#### 4.3.1. Data Collection

**Figure 10:**
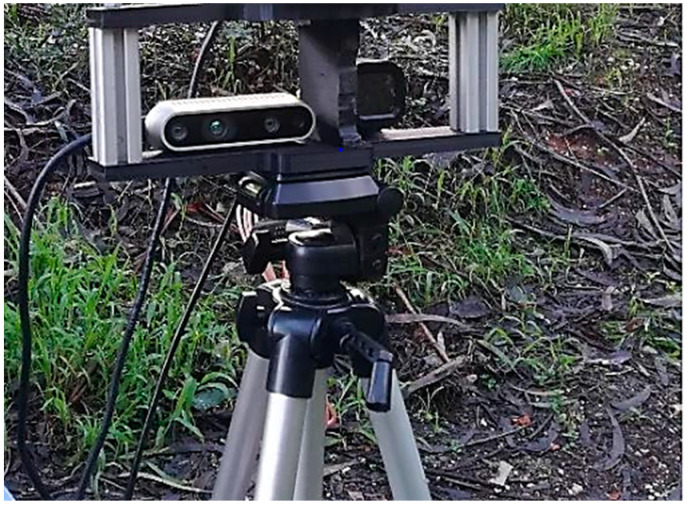
Sensor configuration for data collection.

Data were collected in a forest environment using an RGBD Realsense D435i camera (manufactured by Intel Corporation, Aloha, OR, USA; Hillsboro, OR, USA) and a thermal camera Flir ADK (manufactured by Teledyne FLIR, Wilsonville, OR, USA, EUA) mounted on a support, as shown in Figure 10.

The data collected in a forested area involved recording the output produced by the sensors for 120 s, with the sensor support positioned 5.5 m away from a group of trees and vegetation.

#### 4.3.2. Results Obtained

The YOLOv5s model was trained using a local computer equipped with a six-core AMD Ryzen 3600 processor (manufactured by AMD, Santa Clara, CA, USA), 16 GB of DDR4 RAM, and an NVIDIA GTX 1660 SUPER GPU (manufactured by Nvidia, Santa Clara, CA, USA) with 6 GB of video memory. After the training was completed, the evaluation metric values for each dataset were obtained, and these can be viewed in Table 4.

**Figure 11:**
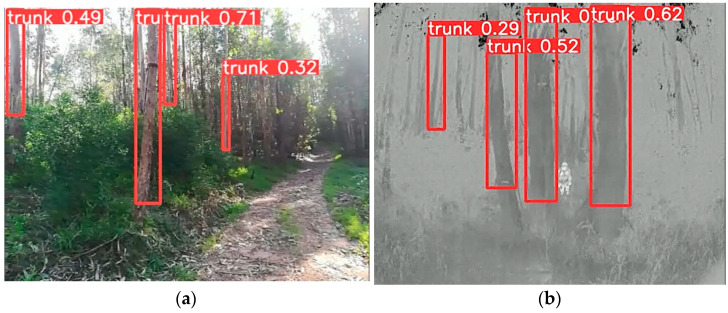
Images taken from the qualitative test regarding the Trees_dataset. (**a**) Image taken by the RGBD camera; (**b**) image taken by the thermal camera.

We can observe that the Trees_dataset has a high mAP:50 score, indicating that the model is highly accurate in detecting tree trunks. This means that, on average, it can recognize the location of tree trunks with great precision, even in scenarios with overlap with other elements in the environment. The mAP50:95 metric demonstrates a good ability to detect tree trunks in different scenarios. The precision metric of 0.901 indicates that the model has a very high rate of correct detection of tree trunks, which is crucial in scenarios where the precise identification of tree trunks is a fundamental aspect. Finally, the recall metric of 0.893 indicates the efficiency of the model in identifying most tree trunks in each environment. Figure 11 shows an example of correct tree trunk detection, where Figure 11a shows an example taken by the RGBD camera and Figure 11b shows an example taken by the thermal camera.

**Figure 12:**
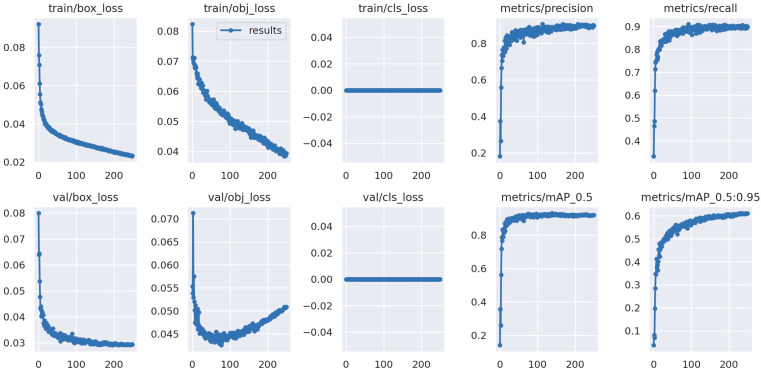
Metrics obtained graphically resulting from the training of the “Trees_dataset” dataset.

In Figure 12, the behavior of all evaluation indices during the training process is visible (in this figure, it is noteworthy that the metrics train/cls_loss and val/cls_loss are zero because the model class was changed to only one, in this case, the trunk).

**Figure 13:**
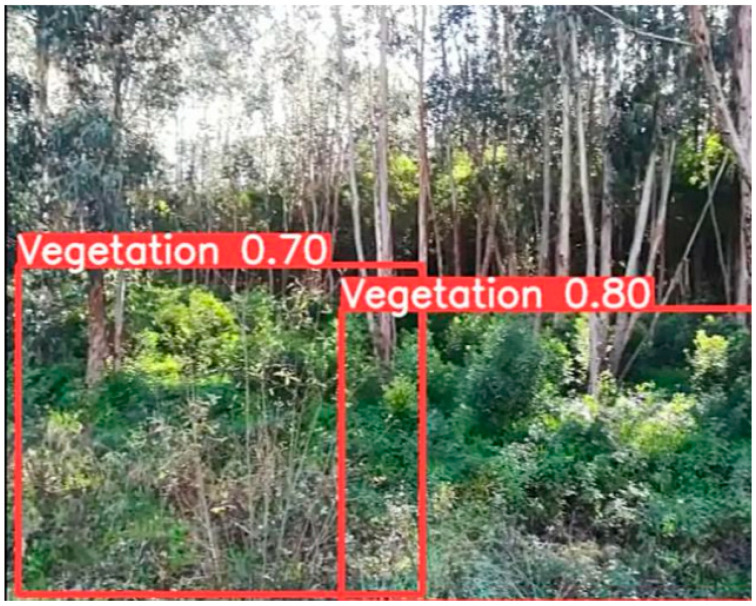
Image taken from the qualitative test related to the Vegetation_detection dataset.

Finally, regarding the Vegetation_detection dataset, the value given by the mAP:50 metric indicates the good accuracy of the model in detecting vegetation. This means that, on average, the model can accurately locate areas with vegetation even when there is overlap with other elements. The value obtained by the mAP50:95 metric indicates that the model may have a somewhat more challenging performance when the overlap with other features (i.e., other elements in the scene) is greater or less than 50%. Regarding the precision metric of 0.796, it indicates that the model has a high rate of correct detection of vegetation, which is extremely important because the model will be able to identify most areas with vegetation. The obtained recall metric indicates that the model can correctly capture most regions with vegetation, suggesting that the model is not missing areas with vegetation. Figure 13 shows an example of the correct detection of areas with vegetation, where the images are provided by the RGBD camera.

**Figure 14:**
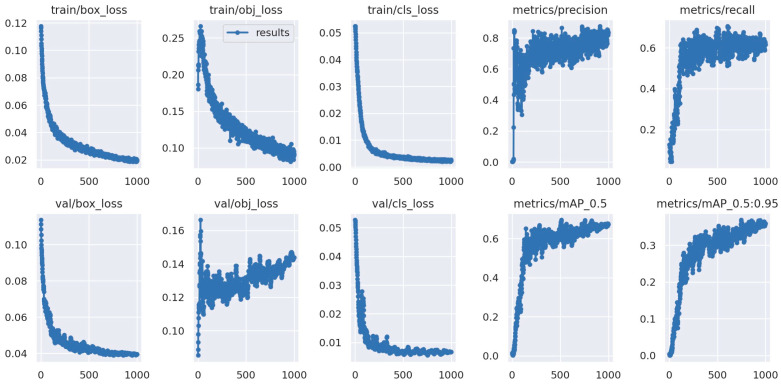
Metrics obtained graphically resulting from the training of the “Vegetation_detection” dataset.

In Figure 14, the training behavior on the “Vegetation_detection” dataset is depicted along with its indicators presented in the graphs.

## 5. Discussion of the Work Conducted

This work presents the development of an integrated vision system for the operation of an autonomous forestry machine, along with a multimodal sensor system called Sentry. The architecture of the vision system was presented, including a comparative analysis and the design logic behind this system. Additionally, two datasets were separately trained for forest environments using the six-core AMD Ryzen 3600 processor (manufactured by AMD, Santa Clara, CA), 16 GB of DDR4 RAM, and an NVIDIA GTX 1660 SUPER GPU manufactured by Nvidia, Santa Clara, CA, USA) with 6 GB of video memory.

Regarding the architecture of the Sentry vision system, it incorporates an object detection algorithm, specifically YOLOv5, chosen for its versatility and effectiveness, making it an asset for the robot’s operation in forest environments. The Sentry system is in a prototyping phase, and its first iteration with the vision system focuses on ensuring safety during the robot’s autonomous operation. A model/algorithm (Figure 6) was created, allowing the measurement of distances to objects (in this case, people) and changing the robot’s state while navigating. The proposed algorithm integrates with the ROS framework and is processed by a minicomputer, in this case, the NVidia Jetson Xavier Nx. The test conducted within the proposed model indicated efficiency in detecting people and their distance from the robot, ensuring safety during the robot’s autonomous operation.

The training of YOLOv5 on two datasets, Trees_dataset and Vegetation_detection, was also presented for the detection of tree trunks and vegetated areas. Based on these datasets, the main goal is to analyze the advantages they can bring during the robot’s navigation. Finally, the results obtained from the training on the proposed datasets have a positive impact on object detection in forest environments. The ability to identify tree trunks is vital because it allows the robot to project them as objects on a specific map to navigate around them if they are in its trajectory. Additionally, vegetation detection is essential for the robot to identify areas with vegetation that need to be cleared for forestry purposes.

## 6. Conclusions and Future Work

The Sentry vision system aims to provide a set of advantages during the autonomous navigation of the robot, such as obstacle detection and distance measurement. As shown in Section 4.2, the detection of people, along with distance measurement, adds an extra layer of security to the robot when it is executing a trajectory.

The trained datasets yielded good results, allowing for the detection and characterization of tree trunks and vegetation, common objects in forest environments.

However, for future work, improvements in the Sentry vision system can be considered. The vision system, composed of RGBD cameras and thermal cameras, needs to be enhanced. Regarding RGBD cameras, in this case, the Intel RealSense D435i, despite its stable performance, it is believed that the application of the Zed 2i camera could be advantageous for the Sentry system, given its good measurement capability of up to 20 m, proving to be a valuable resource. Concerning infrared cameras (FLIR ADK), the detection of some objects can be improved by fusing the data obtained from the RGBD camera and the infrared camera to provide more robustness to the Sentry vision system. The algorithm developed to provide security to the robot during its trajectories can be further enhanced and optimized by integrating it with Visual SLAM (simultaneous localization and mapping) algorithms, thereby enabling the detection and characterization of objects in each environment.

Regarding the trained datasets, future work may consider enhancing robot navigation by integrating the trained datasets into the NVidia Jetson Xavier Nx. For instance, upon detecting tree trunks, the robot could navigate around them, and in the case of vegetation detection, it would recognize the need to clear that area. Additionally, expanding object detection in forest environments to include animals and rocks can further enhance the robot’s ability to distinguish between different objects encountered during its navigation and adapt its behavior to optimize its trajectories.

## Figures and Tables

**Table 1:** Comparative data between Intel RealSense D435I and ZED2i cameras.

Proprieties	Intel RealSense D435i	ZED 2i
Resolution	1280 × 720 (RGB)	2 × 1920 × 1080 (RGB)
Depth range	0.2 m–10 m	0.2 m–20 m
Field of view	91° × 65° × 100°	90° × 59° × 110°
Tracking	6-DoF Pose Tracking	6-DoF Pose Tracking
IMU	Integrated IMU	Integrated IMU
Connectivity	USB 3.0	USB 3.0
Support	Intel RealSense SDK	ZED SDK
Compatibility	Windows, Linux, macOS	Windows, Linux, macOS
Estimated price	EUR 479.00	EUR 879.00

**Table 2:** Number of images in each dataset and their respective distribution.

Dataset	Number of Images	Train/Val/Test Distribution
Trees_dataset	2895	2026/579/240
Vegetation_detection	199	174/17/8

**Table 3:** Values obtained from the test performed on the vision system.

Real Distance (m)	Mean (m)	Variance (m)	Maximum Value (m)	Minimum Value (m)
3	2.855429	0.007638455	3.02	2.66
6	6.675714	0.141206818	7.34	5.7
9	9.778095	0.136692562	11.68	9.17
10	10.61905	0.331344628	11.68	9.5
12	12.17095	0.202512603	13.04	11.41

**Table 4:** Metric values for each dataset.

Train Dataset	mAP:50	mAP50:95	Precision	Recall
Trees_dataset	0.923	0.611	0.901	0.893
Vegetation_detection	0.779	0.478	0.796	0.713

## Data Availability

Data are contained within the article.
